# The impact of perioperative non-steroidal anti-inflammatory drugs use on pleurodesis following thoracic surgery

**DOI:** 10.1093/icvts/ivad107

**Published:** 2023-09-04

**Authors:** Kudzayi Kutywayo, Akolade A Habib, Edward J Caruana

**Affiliations:** Department of Thoracic Surgery, Glenfield Hospital, University Hospitals Leicester, Leicester, UK; Department of Thoracic Surgery, Glenfield Hospital, University Hospitals Leicester, Leicester, UK; Department of Thoracic Surgery, Glenfield Hospital, University Hospitals Leicester, Leicester, UK

**Keywords:** Nonsteroidal, Pleurodesis, Pleural symphysis, Thoracic surgery

## Abstract

A best evidence topic in thoracic surgery was written according to a structured protocol. The question addressed was: in patients who have had {visceral and parietal pleural symphysis}, {do NSAIDs reduce} {the efficacy of pleurodesis}? Sixteen papers were discovered in the search. Of these, 3 human studies were included in the analysis. None showed a significantly higher rate of pleurodesis failure in patients given perioperative NSAIDs. The results from the largest study met criteria for noninferiority. Within the constraints of the study, the results suggest that systemic administration of nonsteroidal anti-inflammatory medication in the perioperative period does not necessarily attenuate effective pleurodesis. However, further study is needed as there is a clear paucity of human-based studies.

## INTRODUCTION

Pleural symphysis usually ensues following thoracic surgical intervention, either by open surgical or thoracoscopic techniques. A variable amount of pleural adhesions also form. These mechanisms lead to the obliteration of the pleural space and prevent the formation of chronic residual spaces. Nonsteroidal anti-inflammatory drugs (NSAIDs) affect inflammation and local immune responses, which are necessary for wound healing and restoration of tissue architecture following trauma or surgery [2]. Their use following thoracic surgery is generally discouraged as it is felt to attenuate pleurodesis which may increase the chances of pneumothorax or effusion formation leading to longer in-hospital stay and further intervention.

To investigate this, a best evidence topic was constructed according to a structured protocol. This is fully described in the ICVTS [1].

## THREE-PART QUESTION

In patients who have had {visceral and parietal pleural symphysis}, {do NSAIDs reduce} {the efficacy of pleurodesis}?

## CLINICAL SCENARIO

You are the on-call registrar on the cardiothoracic ward, and you have been called to review a patient who is day 1 post-surgery.

The patient is a 48-year-old man who underwent a VATS bullectomy and talc pleurodesis for a secondary pneumothorax. He is formerly dependent on heroine and was now on methadone. You have been asked by the nurse looking after him to evaluate his analgesia as he is in significant pain. Due to his background of substance misuse, you are reluctant to give further opioids. The nurse has suggested diclofenac as an alternative. You are however wary of prescribing this as you vaguely recall a colleague cautioning against it. You decide to do a quick literature search to confirm/refute your colleagues’ previous comments before prescribing further pain relief.

## SEARCH STRATEGY

We searched the Medline database using the PubMed interface from 1983 to 2022. The following search query was used: {‘Pleurodesis’} AND {‘nonsteroidal’} AND {pneumothorax}. Limit to English. The search was repeated in the Cochrane Library search from 1983 to 2022 (all dates).

## SEARCH OUTCOME

Twenty-seven papers were discovered.

Nine papers were excluded as they did not address the best evidence question.

Abstracts from 7 papers screened. Two additional papers were discovered on reference review.

A further 6 were excluded—animal-based studies.

Three papers were included in the final full-text analysis (Table 1).

**Table 1:** Best Evidence Papers

Author, date, journal and country Study type (level of evidence)	Patient group	Outcomes	Key results	Comments	Weaknesses
Lizardo *et al.* (2015), J Paed Surg [3] Retrospective review (level III)	*N* = 51 underwent VATS pleurodesis, 26/51 received ketorolac	Length of stay Incidence of residual pneumothorax on discharge Ipsilateral recurrence Follow-up	No difference in the average length of stay between those receiving and those not receiving ketorolac, 11.3 vs 10.9 days (*P* = 0.36) Residual pneumothorax, was identified in 85% of patients who received ketorolac and in 76% of the patients who did not receive ketorolac (*P* = 0.48) There was a 19.6% ipsilateral recurrence rate (10/51) within the study period. All recurrences presented within 3 months of the sentinel procedure. Of the 10 recurrences, 5 patients had received perioperative ketorolac and 5 patients had not (*P* = 1.0) 42 months (24–60 months)	Mechanical pleurodesis: pleural abrasion NSAID: ketorolac	
Rahman *et al.* (2015), JAMA, USA [4] RCT (level II)	Patients undergoing thoracoscopy (*n* = 206) randomized to receive opiates (*n* = 103) versus NSAIDs (*n* = 103) Patients treated conservatively (*n* = 114) randomized to receive opiates (*n* = 57) versus NSAIDs (*n* = 57)	Pleurodesis efficacy at 3 months	Pleurodesis failure occurred in 30 patients (20%) in the opiate group and 33 (23%) in the NSAID group, meeting criteria for noninferiority (difference, −3%; one-sided 95% CI, −10% to infinity; *P* = 0.004 for noninferiority)	Opiate and NSAIDs arm subclassified based on chest tube size (12F vs 24F) NSAID: ibuprofen	
Ben-Nun *et al. (*2011), World J Surg, Germany [5] Retrospective comparative study (level III)	105 patients: 48 in the NSAID group and 57 in the control group	Pneumothorax recurrence rate	The pneumothorax recurrence rate was similar in the 2 groups. 2 patients (3%) in the control group and 1 in the intervention group (2%)	Mechanism of pleurodesis unclear NSAID: diclofenac, etodolac	

CI: confidence interval; VATS: Video-assisted thoracic surgery.

## RESULTS

Lizardo *etal.* [3] in their retrospective review evaluated 51 patients who underwent surgical treatment for primary spontaneous pneumothorax. The period of study evaluated was 4 years.

Lizardo [3] and Ben-Nun’s [5] cohorts were more comparable as they both evaluated patient with pneumothorax. Twenty-six of the 51 had received perioperative intravenous ketorolac. Assignment to the treatment group was clinician dependent.

Pleural abrasion was the method of pleurodesis used apart from 2 patients who received adjuvant talc.

Recurrence rates of pneumothorax in both arms of the study were not dissimilar. This is similar to findings from Ben-Nun [5] who also showed no excess pleurodesis failure rates in a cohort double the size with a longer mean follow-up prior (50 vs 42 months).

Ben-Nun *et al.* [5] conducted a retrospective analysis of 105 patients who had undergone VATS pleurodesis for recurrent spontaneous pneumothorax.

Choice of analgesia postoperatively was physician dependent. Nonrandomized administration of the intervention can potentially bias the results. Without knowing the rationale behind patient selection criteria for giving or not giving nonsteroidals, it is difficult to say with any certainty how this may have biased the results. The same comment can be made in Lizardo’s [3] cohort as the intervention assignment was also clinician dependent.

In the only randomized control trial include in our review, Rahman [4] evaluated in patients with malignant pleural effusion (i) the effect of NSAIDs compared with opiates in the treatment of pleurodesis pain, (ii) the effect of small (12F) chest tubes compared with larger (24F) chest tubes on pain (superiority) and pleurodesis success (noninferiority) in a 2 × 2 factorial. (iii) Evaluation of NSAID on pleurodesis efficacy was a planned subgroup analysis.

The glaring difference between the TIME 1 trial and the other 2 studies was the primary pleural pathology: effusion versus pneumothorax. The mean age in this trial was significantly higher. The mean age in Lizardo *et al.*’s [3] study was 15 years, and the cohort in Rahman *et al.* [4] trial was 71.8 years.

It is interesting to note that in an older demographic with a different indication, use of nonsteroidal met the criteria for noninferiority (difference, −3%; one-sided 95% confidence interval, −10% to; *P* = 0.004 for noninferiority).

Patients treated with opiates had a shorter time to pleurodesis failure compared with those treated with NSAIDs, but this did not reach statistical significance (time to pleurodesis failure: opiates, 48.9 days [standard deviation, 46.3 days]; NSAIDs, 73.0 days [standard deviation, 66.5 days]; hazard ratio, 1.1; 95% confidence interval, 0.7–1.8; *P* = 0.69).

## CLINICAL BOTTOM LINE

Avoiding the use of nonsteroidal anti-inflammatory drugs in the perioperative period following thoracic surgery is commonplace. Previous lab-based animal studies have shown reduced quality of pleurodesis following administration of nonsteroidals [6, 7]. This has vindicated the practice. Following our review, these findings were not corroborated in human studies.

The majority of the studies discovered in our search (6 of 9) were animal-based studies. The authors decided not to include them due to concern regarding the generalizability of the results.

Of the 3 human studies, none showed a significantly higher rate of pleurodesis failure in patients given perioperative NSAIDs. The results from the largest study met criteria for noninferiority. However, this was in patients with a malignant effusion.

It is difficult to make a conclusion with a reasonable external validity considering the paucity of human-based trials.

Within the constraints of the study, the results suggest that systemic administration of non-steroidal anti-inflammatory medication in the perioperative period does not necessarily attenuate effective pleurodesis.

A randomized control study can yield more robust evidence as there is a clear paucity of human-based studies.

**Conflict of interest:** none declared.

## Data Availability

The data underlying this article are available in the article.
